# Eyewitness Lineup Identity (ELI) database: Crime videos and mugshots for eyewitness identification research

**DOI:** 10.3758/s13428-024-02585-z

**Published:** 2025-01-21

**Authors:** Ryan J. Fitzgerald, Eva Rubínová, Eva Ribbers, Stefana Juncu

**Affiliations:** 1 School of Psychology, Sport and Health Sciences, University of Portsmouth, King Henry Building, King Henry I Street, Portsmouth, PO1 2DY UK; 2 Department of Psychology, Simon Fraser University, Burnaby, Canada; 3 Department of Psychology, University of Aberdeen, Aberdeen, UK

**Keywords:** Eyewitness, Face database, Lineup size, Memory, Confidence

## Abstract

There is a long history of experimental research on eyewitness identification, and this typically involves staging a crime for participants to witness and then testing their memory of the “culprit” by administering a lineup of mugshots. We created an Eyewitness Lineup Identity (ELI) database, which includes crime videos and mugshot images of 231 identities. We arranged the mugshots into 6-, 9-, and 12-member lineups, and then we tested the stimuli in an eyewitness experiment. Participants (*N* = 1584) completed six trials of viewing a crime video and completing a lineup identification task. In lineups that included the culprit, the average probability of correction identification was 59.0%, 95% CI [55.9, 62.0]. In lineups that did not include the culprit, the average probability of false alarm was 29.9% [27.8, 32.0]. These outcomes indicate that the ELI database is suitable for eyewitness identification research, and the large number of crime videos would enable stimulus sampling. The database is available for research approved by a research ethics board and can be requested at https://osf.io/vrj3u.

## Introduction

The problem of mistaken identification has been known since the 1970s (e.g., Buckhout et al., 1974; Wells et al., 1979), and yet there is no large collection of standardized stimulus materials available for eyewitness experiments. We created the Eyewitness Lineup Identity (ELI) database as a resource for the scientific community and have made it available for research use.

Several high-quality face databases are already available but were not designed to study eyewitness identification. Face databases typically include 1–3 static images of each identity (e.g., Burton et al., 2010; Chen et al., 2021; Ma et al., 2015, 2020; White et al., 2022). Eyewitness experiments have encoding and test phases, so databases that include only one image per identity would require showing the same image at encoding and test. This creates uncertainty in whether a correct test decision reflects a true recognition of the person or mere recognition of the picture that depicted them (Burton, 2013). This issue is less of a concern when databases contain more than one image per identity, as different images can be presented at encoding and test. However, an eyewitness to a crime would be required to attend to more than just the face of the perpetrator.

The ELI database consists of crime videos and mugshots. In eyewitness identification experiments, the procedure usually begins with a staged crime. Initially, researchers conducted these as live events (e.g., Buckhout et al., 1974; Leippe et al., 1978; Wells et al., 1979). In recent years, however, researchers have typically prerecorded the crime and presented it on video for the witnessing event (Quigley-McBride & Wells, 2021). After the crime has been witnessed, the participant examines a lineup of individuals and decides if one of them is the culprit from the witnessed event. The lineup test also used to be administered as a live procedure or with video recordings (e.g., Brown et al., 1977; Cutler et al., 1989), but photo mugshots have now become the norm. Experimental testing of live lineups indicates they provide no advantage over photo or video lineups (Cutler et al., 1994; Fitzgerald et al., 2018; Rubínová et al., 2021).

Preparing the materials for a lineup experiment is labor-intensive and requires significant resources, planning, and pilot-testing. To avoid floor and ceiling effects, pretesting may be required to find the right amount of exposure to the culprit at the witnessing event and the right degree of similarity between the culprit and the fillers in the lineup (Wells & Penrod, 2011). Furthermore, to reduce the risk of stimulus-specific effects and increase the generalizability of the findings, it is generally recommended to use multiple sets of culprits and lineups (Wells & Windschitl, 1999). One of the goals of the ELI database was to facilitate stimulus sampling, and thus we recorded crime videos featuring over 200 identities. Although some researchers have shared crime videos for a perpetrator or two (e.g., Kruse & Schweinberger, 2023; Oriet & Fitzgerald, 2018; Schooler & Engstler-Schooler, 1990), we are not aware of any existing databases that include crime videos for so many individuals.

We requested consent from the ELI actors to share the database with other researchers and to include the mugshot images in academic publications. To fully understand a lineup experiment, it is necessary to see the lineups that were tested. Nevertheless, most researchers do not make their lineup materials available. This is often because they have not obtained consent from the actors to share their images. To enable the dissemination of lineups made with the ELI database, we obtained consent to share the images with other researchers and in journal publications.

After describing the details of the database, we report findings from an eyewitness identification experiment with lineups constructed from the ELI database. We designed the experiment to test all the identities that could potentially be included together in a lineup. This enabled us to generate hit rates and false-alarm rates at the level of the identity. We also used data from the experiment to determine which identities are most likely to be confused with one another. These data can inform stimulus selection for future experiments.

### Eyewitness Lineup Identity (ELI) database

Approval to create the database was obtained from the Science Faculty Ethics Committee at the University of Portsmouth. The procedures adhere to the tenets of the Declaration of Helsinki.

#### Identities

The ELI database includes crime videos and lineup images of 231 identities. The crime videos and lineup images were recorded on the same day^1^, and all participants were recorded in Portsmouth, England. Due to experimenter error, 25 identities did not complete the questionnaire for self-reporting demographic details. In the remaining sample, most identified as female (128 female, 73 male, five prefer not to say), young adult (*M* = 22.74, *SD* = 6.76, range = 18–74 years; 4 prefer not to say), and White (131 White, 22 Black, 19 Mixed, 11 East Asian, six South Asian, six Middle Eastern, three Hispanic/South American, three Southeast Asian, and one Pacific Islander, four prefer not to say).

#### Crime videos

Crime videos were recorded using a Cannon EOS 750D (W) 24.2 megapixel digital camera. Crime videos up to Identity 153 are set in a hallway (one of two hallway locations) and depict an individual walking up to a bag placed on a chair, looking around, taking a laptop or tablet from the bag, and walking away. Crime videos from Identity 154 are set in a room and depict an individual opening the door, walking up to a bag placed on a table, looking around, taking a tablet from the bag, and walking away through the door. The crime videos have no sound and include no other people. The videos depict the culprit at a distance of approximately 13 ft (4 m) from the camera. The average length of the videos is *M* = 26 s (*SD* = 4). The video includes a frontal view of the face (for approximately 3 s) as well as profile views. Additional crime videos of each identity were recorded, but they have not been systematically tested and are not included as part of the database. Two exceptions are for identities #172 and #252. The standard versions with the single 3-s frontal view could not be included because they depicted a bystander, so we included a longer version for these identities instead. The longer videos for #172 and #252 are 40 and 30 s in duration, respectively, and include three frontal views of the face of ~3 s.

#### Lineup mugshots

Fifteen-second mugshot videos were created with a recording booth on loan to the first author from the VIPER group (Video Identification Parade Electronic Recording). VIPER is based at West Yorkshire Police and provides eyewitness identification products and services for approximately half of all police forces in the United Kingdom. These videos were recorded with the same model of digital camera that was used for the crime videos (Cannon EOS 750D). During the 15-s recording, the experimenter instructed the participant to face straight (0–4 s), turn left (4-s mark), turn right (7-s mark), and face straight (10-s mark). Photo mugshots were extracted from the videos. In the eyewitness experiment, we only tested frontal photo mugshots, and only these photos are included in the database.

#### Use of ELI database

To access the ELI database, visit https://osf.io/vrj3u. The database stimuli are organized into two folders, one for crime videos and one for mugshot images. Each identity is assigned a number (see Figs. 4, 5, 6, 7, 8, 9, 10, 11. Identity numbers in Table 2 correspond with the numbers in Figs. 4, 5, 6, 7, 8, 9, 10, 11, 12, 13, 14, 15, 16, 17, 18 and 19 in the Appendix), and this number is the file name of each identity in the crime video and mugshot folders. The OSF page also includes datafiles that include information about the identities, including demographics, performance outcomes (hit and false-alarm rates in the experiment reported below), and a list of the eight ELI identities that were most often confused with the identity or judged to be similar (for more information, see Datafiles section below).

Use of the database is subject to the following conditions:

1. The images and associated information must be used exclusively for the purpose of academic research that has been approved by a research ethics committee.
2. The images and associated information must be stored securely.
3. The images and associated information must not be used for any form of commercial activity.
4. The database must not be distributed to any third party.
5. Any research disseminated using this database will cite the current manuscript.
6. Images may be included in the academic journal articles but must be accompanied by the following acknowledgement:

This research used images from the Eyewitness Lineup Identity database (Fitzgerald et al., 2025). The people depicted in the database are actors, not actual culprits or lineup members in real criminal cases. Mugshots in the database were created with a recording booth on loan from the Video Identification Parade Electronic Recording (VIPER) Bureau, West Yorkshire Police, England. Images in the database were not quality assured by the VIPER Bureau, and the authors accept full responsibility for their quality.

#### Eyewitness identification experiment

We initially tested the database with undergraduate students who participated in an eyewitness identification experiment. Our objectives were (a) to measure the difficulty of the crime videos, (b) to obtain descriptions and lineup performance metrics for the identities, and (c) to measure the number of plausible options in a set of lineups. We constructed lineups of different sizes, which also allowed us to compare performance across 6-, 9-, and 12-member lineups. Thus far, a limited number of lineups have been constructed from the ELI database and tested in eyewitness identification experiments (Kruisselbrink et al., 2023; Fitzgerald et al., 2023). In the current eyewitness experiment, we tested 72 lineups and showed crime videos with 174 of the ELI identities to provide a more comprehensive test of the database.

## Method

Approval was obtained from the Research Ethics Board of Simon Fraser University. The procedures used in this study adhere to the tenets of the Declaration of Helsinki.

### Participants

The stimulus materials were tested on 1587 undergraduate students enrolled in 1st or 2nd year psychology courses at Simon Fraser University. All participants received partial course credit in exchange for their participation. The sample was 69.6% women, 28.3% men, 1.0% other, and 1.1% prefer not to say. Ages ranged from 17 to 43 years (*M* = 19.2, *SD* = 2.2; *n* = 54 prefer not to say). Most participants identified their ethnicity as White (29.8%), South Asian (22.9%), or East Asian (22.0%). Additional ethnicities reported included Southeast Asian (6.9%), West Asian (3.5%), Latin American (1.8%), Black (1.7%), Arab (1.6%), Indigenous (.6%), and “Other” (6.9%). The remaining 2.4% did not report their ethnicity.

Participants viewed six crime videos and completed six lineup identification procedures, yielding an initial dataset of 9522 trials. If participants failed the attention check (explained below), we reviewed their description of the culprit for that trial. For a description to be classified as sufficient, it needed to include more than one accurate descriptor. If the description was insufficient, or if they used the description form as an opportunity to report a problem with the video, we excluded the trial. We also closely inspected the trials of all participants who failed multiple attention checks and excluded any of their trials that were judged to have insufficient descriptions. We excluded all trials from three participants who failed all six attention checks. A total of 69 trials were excluded, resulting in a final dataset of 9453 trials from 1584 participants.

### Mugshot lineups

We tested 72 lineups. The first author constructed the lineups by first organizing the identities into pools according to their match to a broad description (i.e., sex, age, ethnicity, and hair) and then further refining the pools into 6-, 9-, and 12-member lineups. The descriptions were sufficiently broad to result in heterogenous pools of identities who varied in resemblance with one another. From the pools, lineups were constructed by removing lineup members who appeared to stand out from the others because of a particular feature. For example, one lineup was constructed by removing people whose hair was less curly than others in the pool. To test as many identities as possible, we sometimes included people in the lineups who did not fully match the description. For example, we sometimes included South Asian or Southeast Asian people in lineups with East Asian people.

We constructed different numbers of lineups per pool, depending upon whether a reasonable 12-person lineup could be constructed (*n* = 4) or not (*n* = 7). The four pools that were large enough to construct 12-member lineups are depicted in Figs. 4, 5, 6, 7, 8, 9, 10, 11. Identity numbers in Table 2 correspond with the numbers in Figs. 4, 5, 6, 7 and 8, 9, 10, 11 (see Appendix). For the six pools depicted in Figs. 12, 13, 14, 15, 16 and 17, we constructed three six-member lineups. For the last pool, depicted in Figs. 18 and 19, we constructed six six-member lineups.

In the first four pools, we started by constructing 12-member lineups and then used those as a basis for constructing 6- and 9-member lineups. This enabled us to manipulate lineup size while controlling for the identities, who all appeared in 6-, 9-, and 12-member lineups. Note, however, that we did not fully counterbalance identities across the 6-, 9-, and 12-member lineups. Rather, from each 12-member lineup, two nine-member lineups and three six-member lineups were constructed. In the first nine-member lineup, the first nine identities were selected (according to their number in the database). In the second nine-member lineup, the last nine identities were selected. The three six-member lineups were constructed with identities 1–6, 4–9, and 7–12. We opted for this approach, rather than fully counterbalancing or randomly selecting lineup members from the pools, to limit the number of lineups and maximize the number of datapoints per lineup. This allowed us to compute lineup fairness estimates for the 72 lineups (see Results).

### Crime videos

We used crime videos of 174 ELI identities in the experiment. Some identities in the database were excluded because they were too distinctive (e.g., purple hair) to be included in the lineups we constructed.

Prior to the lineup on a given trial, a crime video was randomly selected from that lineup’s pool. Depending on whether the identity in the selected crime video was also included in the lineup or not, this produced trials that sometimes contained the culprit (culprit-present condition) and other times contained only innocent people (culprit-absent condition). This method of manipulating the presence of the culprit in the lineup is a variation of the single lineup paradigm (Oriet & Fitzgerald, 2018). The random selection of culprits created variability in the similarity between the culprit and the lineup members. This reflects that in real cases, the similarity between an innocent suspect and the culprit would vary (Wells & Penrod, 2011), and fillers would be matched to the innocent suspect rather than to the culprit (Clark & Tunnicliff, 2001). Due to variations in the size of the pools, our method of selecting crime videos initially produced variability in the base rate of culprit-presence. After some initial testing, we reprogrammed the experiment to better approximate a 50% base rate of culprit-presence. Crime videos for six identities (160, 161, 162, 163, 164, 170) were temporarily missing and were not included in the experiment, but they have since been recovered and are included in the database.

### Attention check

At the end of each crime video, a target picture of an object such as a fruit or plant appeared for 4 s. As an attention check, participants were tested on their recognition of the picture using a multiple-choice attention test that included the target picture and five filler pictures that did not appear in the video. The five filler pictures were chosen to be semantically and visually unrelated to the target picture to minimize the risk of error by participants who attended to the video.

### Procedure

The study was advertised as an eyewitness identification experiment. Participants were instructed that they would view crime videos and that their task was to remember the person who appeared in the videos for a subsequent lineup identification procedure. They were also warned that the person from the video may or may not be in the lineup.

Each participant completed six eyewitness identification trials. On Trials 1–4, participants were randomly assigned to complete 6-, 9-, or 12-member lineups (a between-subject manipulation). These trials always proceeded in the following order: White young adult women with dark hair, White young adult men with dark hair, Black young adult women, and, finally, young adult women of multiple races. The mugshot images used on the first four trials are reported in Figs. 4, 5, 6, 7, 8, 9, 10, 11. Identity numbers in Table 2 correspond with the numbers in Figs. 4, 5, 6, 7, 8, 9, 10 and 11 in the Appendix. On Trials 5–6, participants completed lineup tasks that always included six mugshots and were constructed from two of the remaining pools: East Asian men, East Asian women, men of multiple races, White men with blonde hair, White men with facial hair, White women with black hair, and White women with blonde hair (see Appendix, Figs. 12, 13, 14, 15, 16, 17, 18 and 19). We opted not to randomize the order of trials for identity groups because we were able to make better lineups for some identity groups over others, and we prioritized testing of the higher-quality lineups. Although this prevented us from distinguishing between trial order effects and identity group effects, we decided not to randomize the order to avoid trials with the low-quality lineups from affecting performance on trials with the higher-quality lineups.

The sequence of each trial was: (1) crime video, (2) attention check, (3) description of the culprit, (4) 1-min distractor task, (5) lineup task, (6) confidence rating. For the description, participants were given an open-ended response option and were instructed to report the culprit’s sex, race/ethnicity, build, age, hair, and any distinctive features. For the distractor task, participants were instructed to take a break or play the computer game “Snake”. The lineup task had two parts: participants were first instructed to select the lineup member who was the most similar to their memory of the person from the video and then they were asked, “Is this the person from the video?”. This is known as the elimination lineup procedure (Pozzulo & Lindsay, 1999). Lineup member position was counterbalanced within each lineup. The confidence rating was given on an 11-point scale, ranging from 0–100%.

### Datafiles

Two datafiles are available at https://osf.io/vrj3u. One is labeled “Eyewitnesses” and contains data from the 9453 trials of the eyewitness identification experiment. This file includes the lineup task responses (description of the culprit, identification response, confidence), stimulus details, and participant demographics for each trial of the eyewitness experiment.

The second file is labeled “Identities”^2^ and reports the following: (1) Response data from the eyewitness experiment aggregated at the level of the identity, such as how often participants saw the identity in a crime video and then (a) correctly identified them from a culprit-present lineup or (b) mistakenly identified a different person from a culprit-absent lineup, i.e., a false alarm. (2) Self-reported gender, age, and race of the identities. The self-reports of gender and age were coded by the first author. The self-reports of race were coded by the first and second authors into one of the following categories: Black, East Asian, Hispanic, Middle Eastern, Pacific Islander, South Asian, Southeast Asian, White, Mixed, or if no race was reported it was coded as NA. (3) The eight most similar identities in the database for each identity, ranked via a similarity index.

The similarity index was created in three steps. In Step 1, we produced a false-alarm rate for each identity. The false-alarm rate indicated how often seeing one identity in a crime video led to misidentifying another identity in a culprit-absent lineup. We calculated this rate by identifying all the culprit-absent trials associated with a given identity as the culprit and determining how often each other identity was mistakenly identified as that culprit. For example, on trials that depicted Identity A as the culprit, we calculated a false-alarm rate for Identity B by isolating all trials that showed the crime video for Identity A followed by a culprit-absent lineup that included Identity B. Then we divided the number of times Culprit B was misidentified by the number of trials.

In Step 2, we produced a “most-similar” rate. For this, we looked at how often participants selected a lineup member at Stage 1 of the identification task; namely, when they were forced to choose which lineup member was the best match to their memory. Note that this measure indicates lineup members who were chosen as the most similar to the culprit but who were also not identified in Stage 2 of the identification task, which asks if the most similar person is the culprit. The most-similar rate was calculated by dividing the number of times they were selected as the most similar lineup member at Stage 1 (and not misidentified at Stage 2) by the number of times the identity appeared in a culprit-absent lineup.

In Step 3, we applied an algorithm to derive a similarity rank. The algorithm was designed so that the top-ranked identity was the one with the highest false-alarm rate for a given culprit, followed by the identity with the second highest false-alarm rate, etc. If there were fewer than eight identities that elicited false alarms, the algorithm considered most-similar rates (higher rate = higher rank). If there were fewer than eight identities that elicited most-similar responses, the algorithm considered the number of appearances in the culprit-absent lineup (fewer appearances = higher rank, based on the inference that frequent appearances that elicited no false alarm or no most similar responses were indicative of lower similarity). The identities file specifies the top eight ranking identities for each identity that was used as the culprit in a crime video. There was not typically enough false alarms or most-similar responses to produce rankings that would be informative beyond the top eight.

## Results

**Fig. 1:**
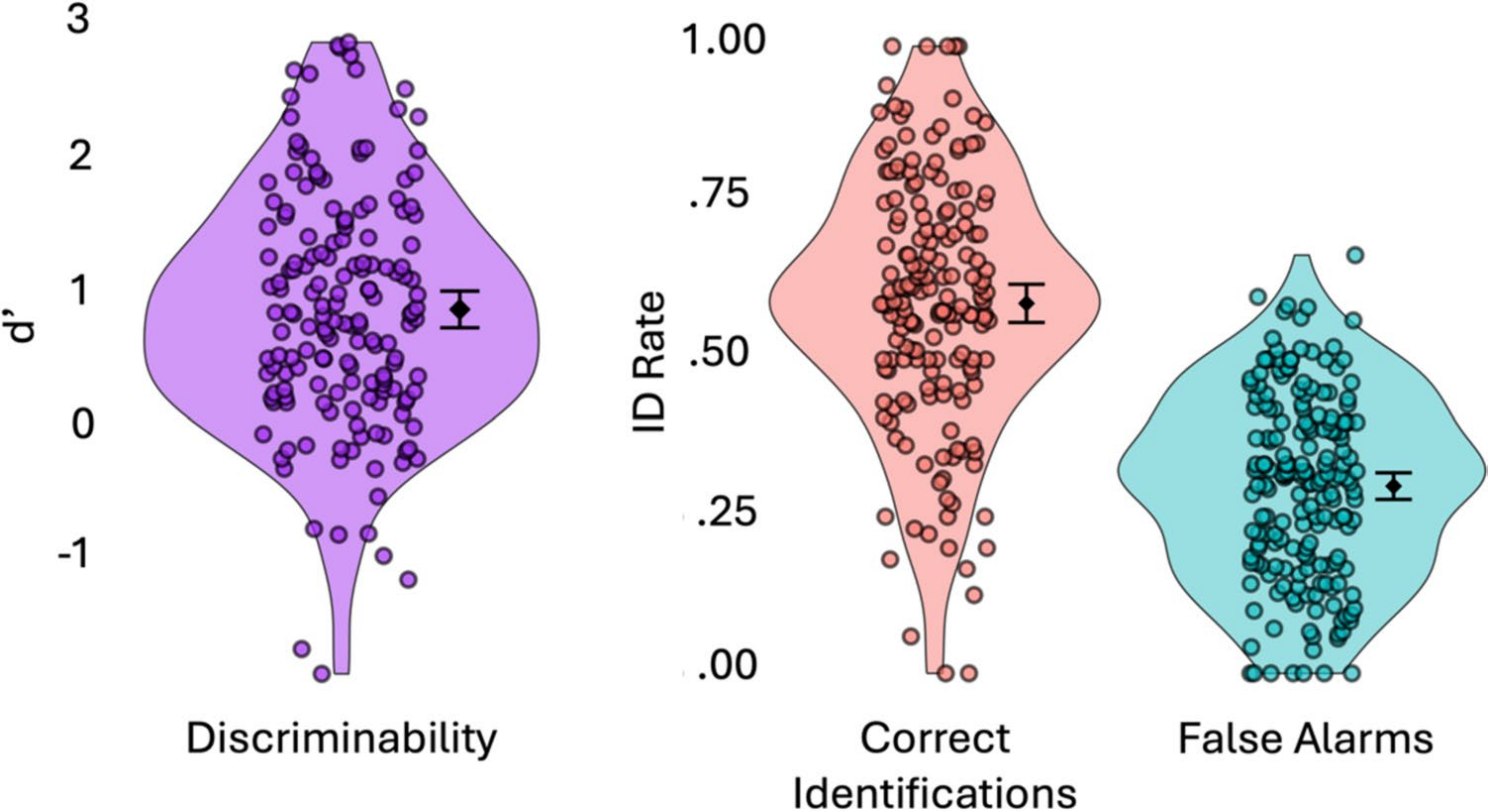
Outcomes for identities that appeared as culprits in the eyewitness experiment. *Note.* Each *point* shows the outcome when a particular identity appeared as the culprit. *Diamonds* are means. *Error bars* are 95% CIs

Figure 1 reports correct identifications, false alarms, and *d’* for the identities*.* Throughout the results we report effect sizes for all pairwise comparisons with 95% CIs in brackets. Each identity appeared as the culprit on average 48.9 times (*SD* = 18.4), but crime videos were randomly selected on each trial, and some identity groups were larger than others, so there is substantial variability in cell counts across identities. For culprit-present lineups, there was an average of 25.5 (*SD* = 14.7) trials per identity. For culprit-absent lineups, there was an average of 25.7 (*SD* = 12.3) trials per identity.

Culprit-present lineups were used with 173 identities, yielding correct identification probabilities ranging from 0 to 100%, *M* = 59.0, 95% CI [55.9, 62.0]. These correct identification rates were calculated by dividing the number of times that identity was identified as the culprit by the number of culprit-present lineup trials with that identity as the culprit. The crime videos for two identities resulted in 0% hit rates (*n* = 19 for #71 and *n* = 9 for #196), indicating that their appearance in the crime video was not a good match to their appearance in the mugshot.

False-alarm probabilities were obtained for 190 identities and ranged from 0% to 66.7%, *M* = 29.9, 95% CI [27.8, 32.0]. These probabilities apply to culprit-absent trials with the identity as the culprit. The rate was calculated by dividing the number of times that any lineup member was identified by the number of trials. Thus, a false-alarm probability reflects how often an eyewitness saw the identity in the crime video and then proceeded to mistakenly identify a different identity from a culprit-absent lineup.

The discriminability score, *d’*, was computed using the formula *d’* = *z*(correct identification rate) – *z*(false-alarm rate). For rates 0 or 1, a log-linear correction was applied (i.e., we adjusted frequencies in the rate calculation by 0.5 in the numerator and 1.0 in the denominator; Hautus, 1995). A high *d’* score indicates that an identity is memorable or distinctive relative to the other identities, whereas a low *d’* score indicates that an identity is harder to remember or more easily confused with other identities. In the sample of 165 identities who appeared in both culprit-present and culprit-absent lineups, *d’* ranged from – 1.83 to 2.82, *M* = 0.85, 95% CI [0.72, 0.99]. Note that negative *d’* values are possible because to make a hit, the participant needs to identify the target from a group of 6-, 9-, or 12-lineup members. This results in relatively low hit rates. By contrast, the false-alarm rates were relatively high because we included false alarms of any lineup member from the culprit-absent lineup in our calculation.

### 6 vs. 9 vs. 12-member lineups

**Fig. 2:**
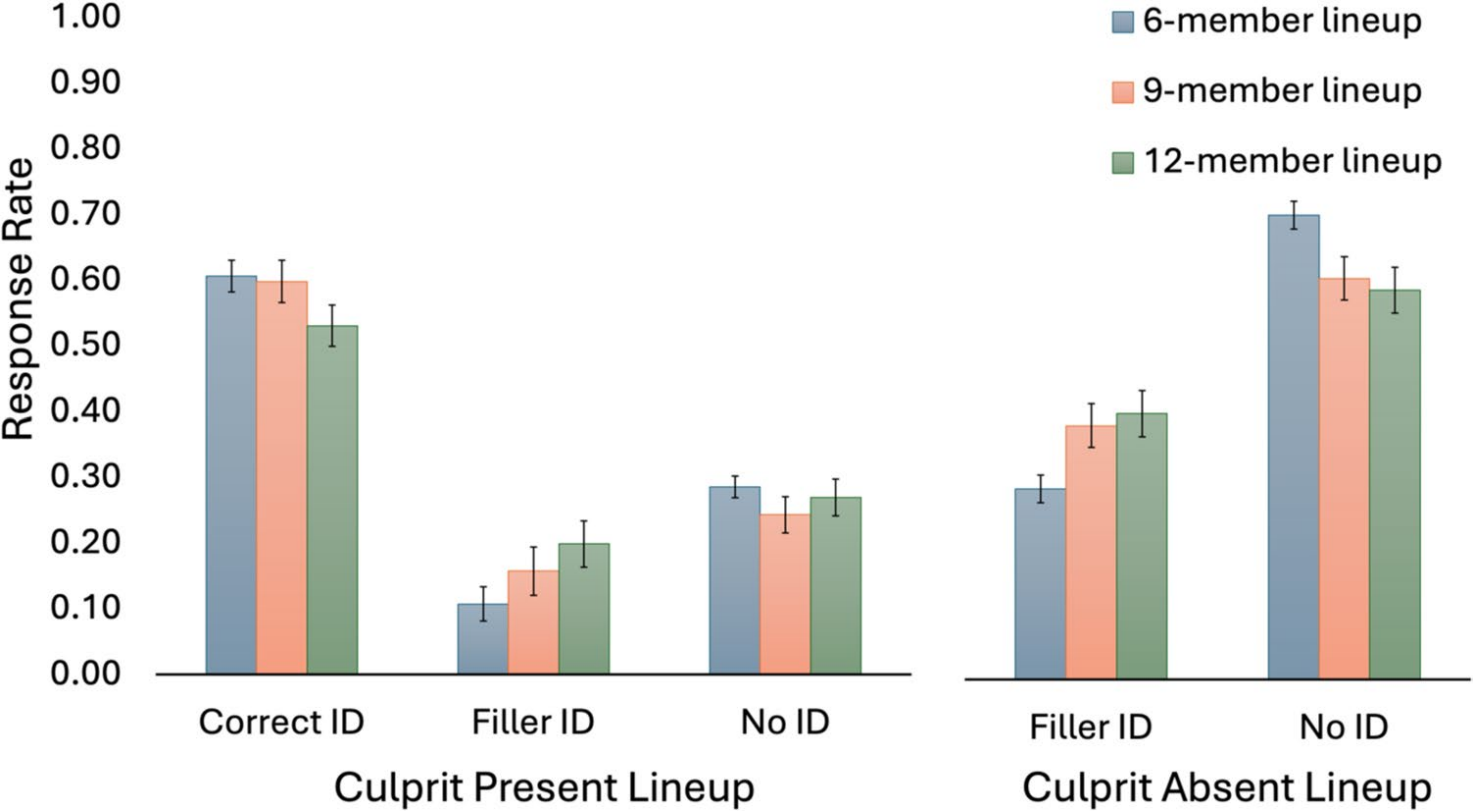
Lineup response rates in 6-, 9-, and 12-member lineups. *Note.* Results of first 4 trials of the eyewitness identification experiment (6306 trials). Error bars are 95% CIs

Figure 2 depicts lineup outcomes for the three lineup size conditions. Lineup size was only manipulated on the first four trials, and thus we only included data for these trials to assess for differences across the lineup size groups. Disaggregated data for the 48 lineups used on the first four trials are reported in Table 1 in the Appendix.

#### Lineup fairness

Effective size is a measure of lineup fairness that reflects the number of plausible lineup members (Malpass, 1981). We estimated effective sizes from the distributions of choices in the culprit-absent lineups (Quigley-McBride & Wells, 2021; Smith et al., 2021). Using Tredoux’s (1998) formula, the average effective sizes of 6-, 9-, and 12-member lineups were 4.00 [3.74, 4.25], 5.27 [4.59, 5.95], and 6.62 [5.23, 8.01], respectively. A one-way ANOVA indicated the increase in effective size across the lineup size conditions was significant, *F*(2, 45) = 19.52, *p* < .001.

#### Eyewitness accuracy

Eyewitness accuracy refers to the accuracy of all identification decisions. Accurate responses included correct identifications on culprit-present lineups and nonidentifications on culprit-absent lineups. All other responses were incorrect.

A generalized linear mixed model was used to predict eyewitness accuracy with lineup size and culprit presence as fixed factors and random intercepts for subjects and lineups. We used successive difference contrast coding to compare culprit present vs culprit absent lineups, and for lineup size, to compare 6- vs. 9-member lineups and 9- vs. 12-member lineups. To be able to evaluate the 6- vs. 12-member lineup comparison, we computed a second model with this contrast.

Accuracy was higher for culprit-absent than culprit-present lineups: 65.9 vs. 58.4%, respectively, *OR* = 1.32 [1.18, 1.48], *z* = 4.75, *p* < .001. Accuracy was also higher on six-member lineups (65.8%) over both nine-member lineups (60.5%), *OR* = 1.28 [1.04,1.58], *z* = 2.32, *p* = .020, and 12-member lineups (56.2%), *OR* = 1.55 [1.22, 1.97],* z* = 3.57, *p* < .001. The difference between nine-member and 12-member lineups was not significant, *OR* = 1.21 [0.93, 1.57], *z* = 1.41, *p* = .159. A significant interaction between culprit presence and lineup size in the comparison of six- and nine-member lineups indicated that the increase in accuracy for six-member lineups was greater in culprit-absent lineups (6 = 70.9% vs. 9 = 61.2%) than in culprit-present lineups (6 = 60.7% vs. 9 = 59.9%), *OR* = 1.52 [1.16, 1.99], *z* = 3.05, *p* = .002.

#### Estimating innocent suspect identifications

We applied a correction to the false-alarm rate in culprit-absent lineups to see if lineup size affected the estimated risk to an innocent suspect. The best practice for constructing police lineups is to include only one suspect in the lineup and for all remaining lineup members to be known-innocent fillers (Wells et al., 2020; Wells & Turtle, 1986). Assuming the lineup is constructed using this single-suspect model and there is no bias against the suspect, then the risk to the suspect can be estimated by dividing the overall false-alarm rate in the culprit-absent lineup by the number of lineup members. This is known as the nominal size correction. When this correction was applied, the estimated rate of innocent suspect identifications was 4.9% [3.8, 5.9], 4.3% [2.9, 5.7], and 3.4% [2.0, 4.7]), respectively for 6-, 9-, and 12-member lineups. The highest innocent suspect ID rate was not significantly different from the lowest one, *z* = 1.65, *p* = .09.

#### Positive predictive value (suspect identification accuracy)

We computed the positive predictive value (PPV) of the three lineup sizes. PPV ranges from 0 to 100% and reflects the probability that the suspect is guilty if the eyewitness identifies them from the lineup (Mickes, 2016). The nominal-size-corrected estimate of PPV was calculated using the formula: PPV = correct identification rate/(correct identification rate + [culprit-absent lineup false-alarm rate/nominal lineup size]). To account for unequal sample sizes across lineup size conditions, we used the sample sizes for 12-member lineups as the reference group and scaled the sample sizes of the 6- and 9-member lineups to be equivalent. The PPV rates were all very similar: Size 6 = 92.3% (*n* = 525), Size 9 = 93.4% (*n* = 514); and Size 12 = 94.7% (*n* = 453). The difference between the two most distant PPVs was not significant,* z* = 1.51, *p* = .131. We also estimated PPV by dividing the false-alarm rate by the lineup’s effective size, rather than its nominal size, and again found no significant effect of lineup size on PPV.

**Fig. 3:**
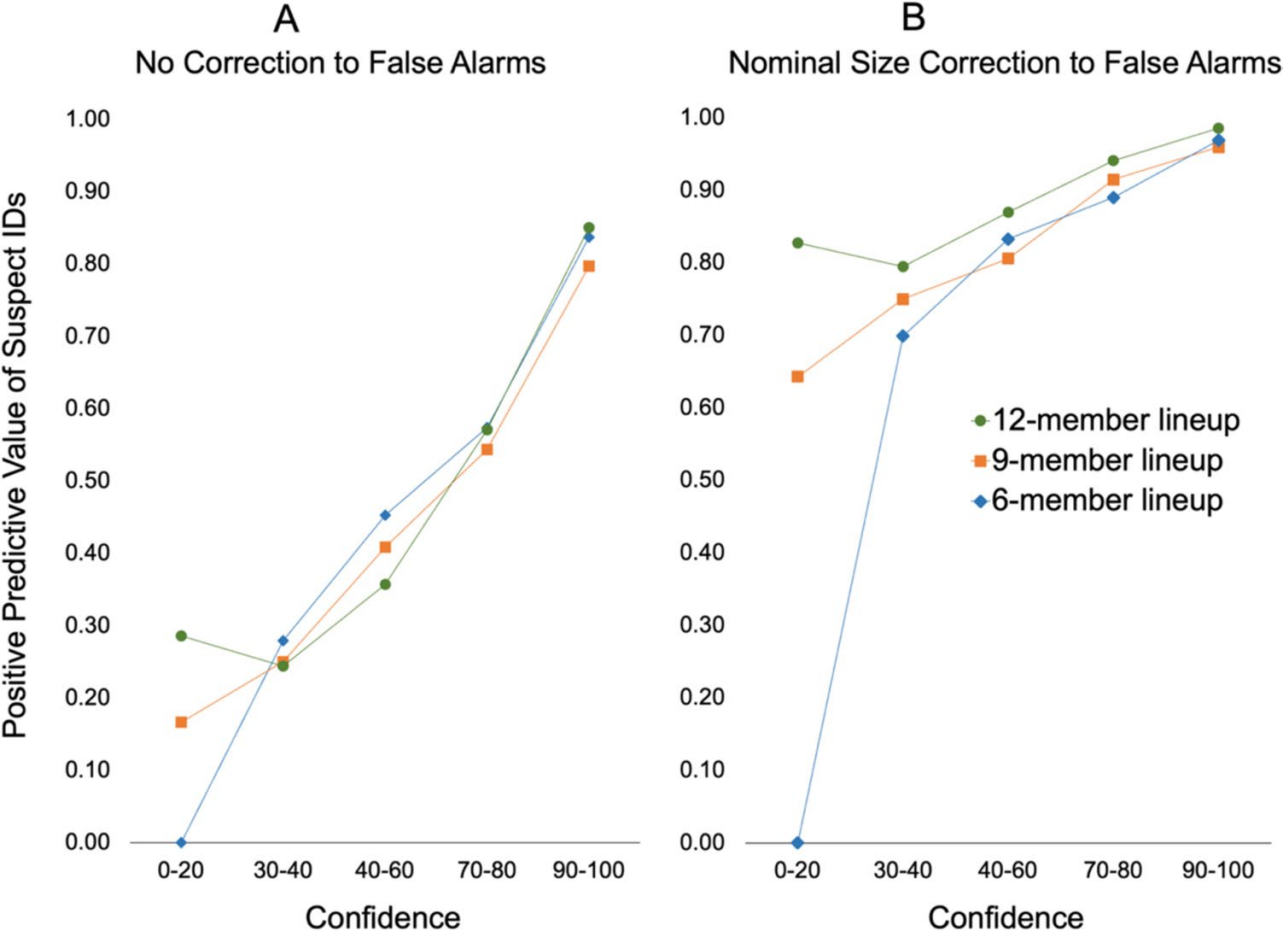
Positive predictive value of suspect identifications (IDs) in 6-, 9-, and 12-member lineups

**Fig. 4:**
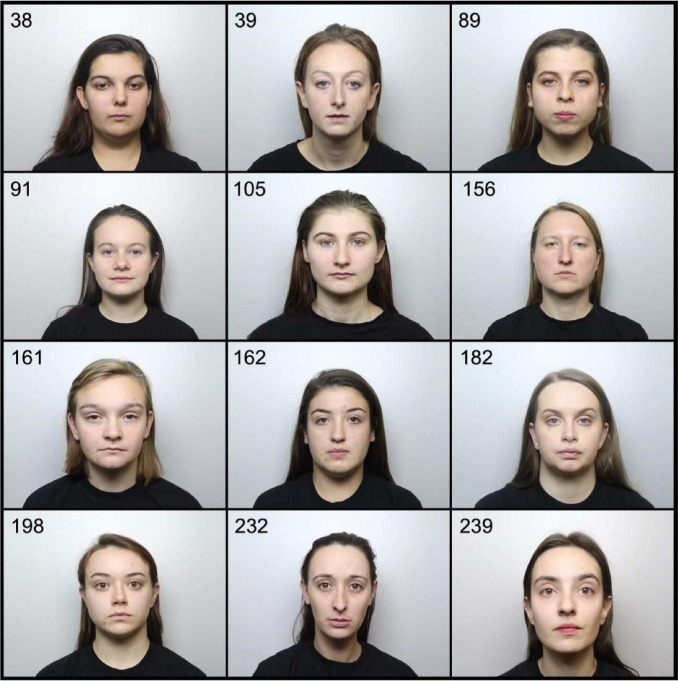
White female brunette 1

**Fig. 5:**
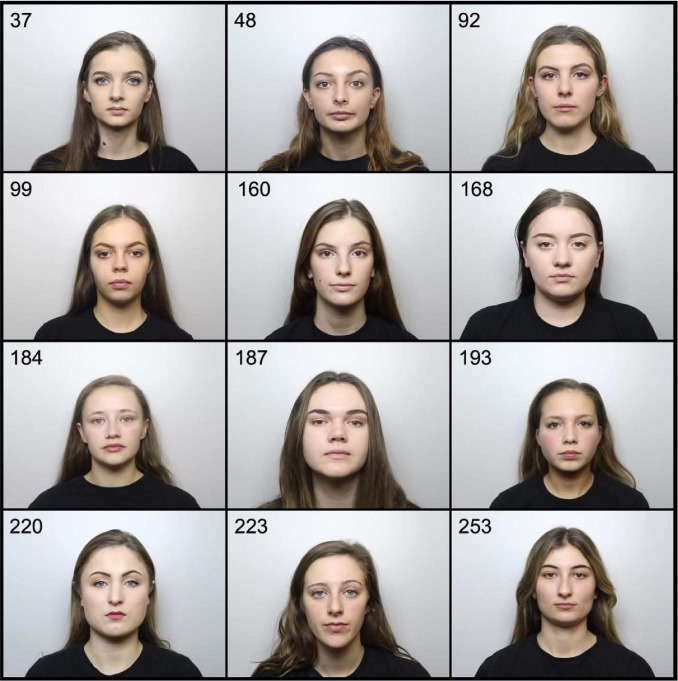
White female brunette 2

**Fig. 6:**
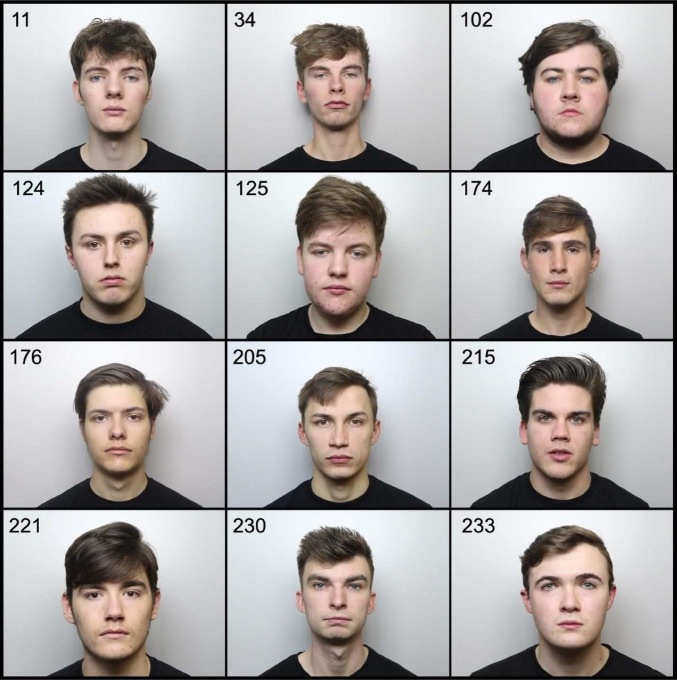
White male brunette 1

**Fig. 7:**
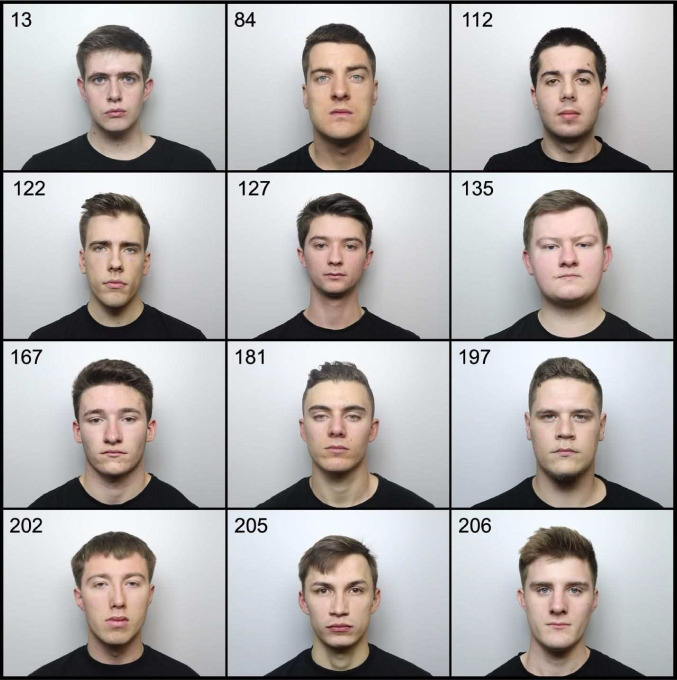
White male brunette 2

**Fig. 8:**
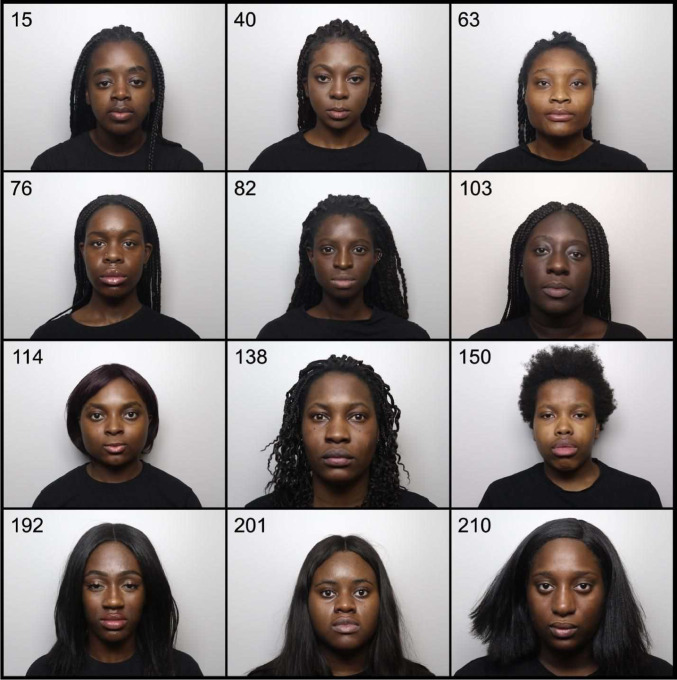
Black female 1

**Fig. 9:**
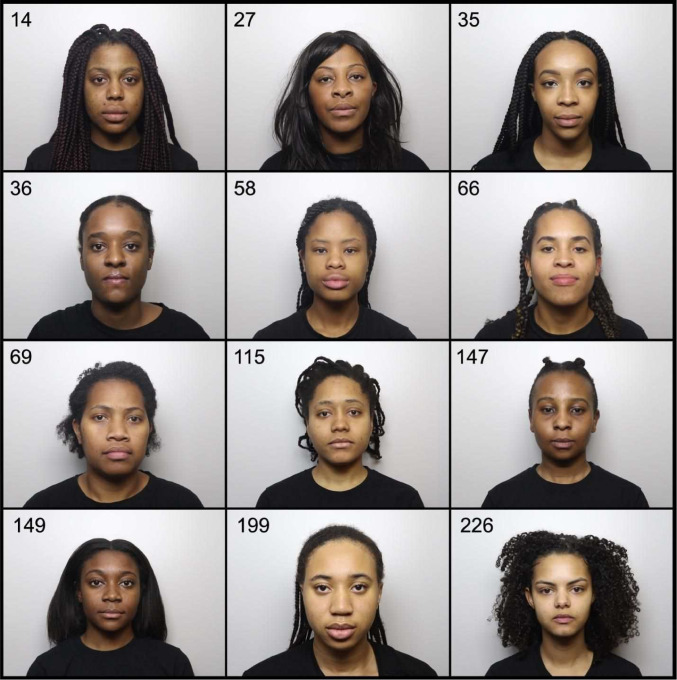
Black female 2

**Fig. 10:**
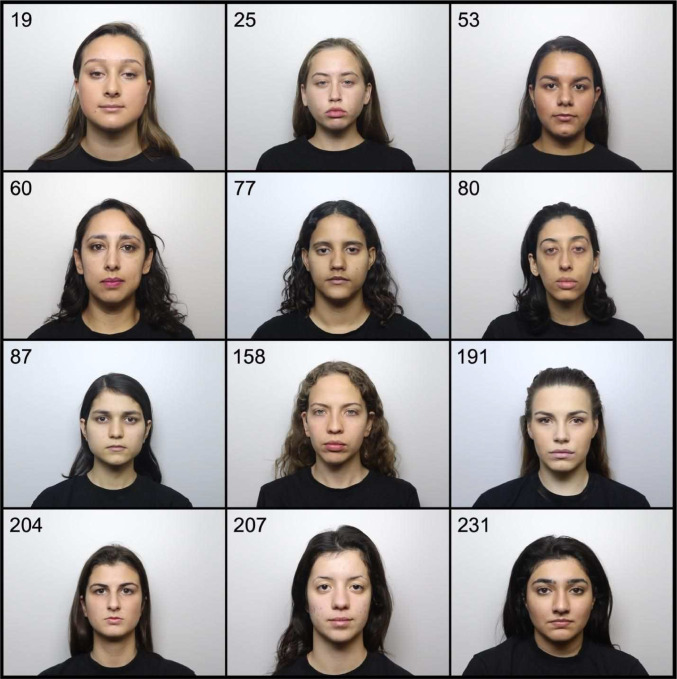
Multiple races female 1

**Fig. 11:**
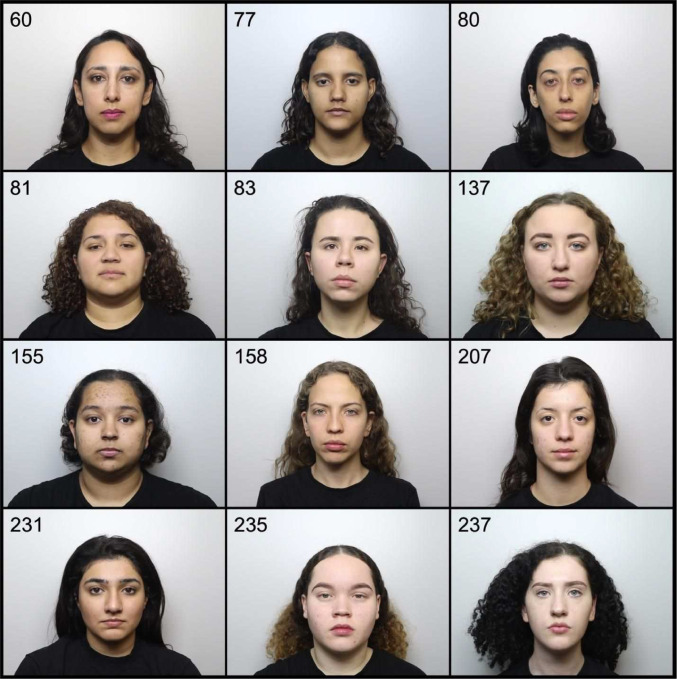
Multiple races female 2

**Fig. 12:**
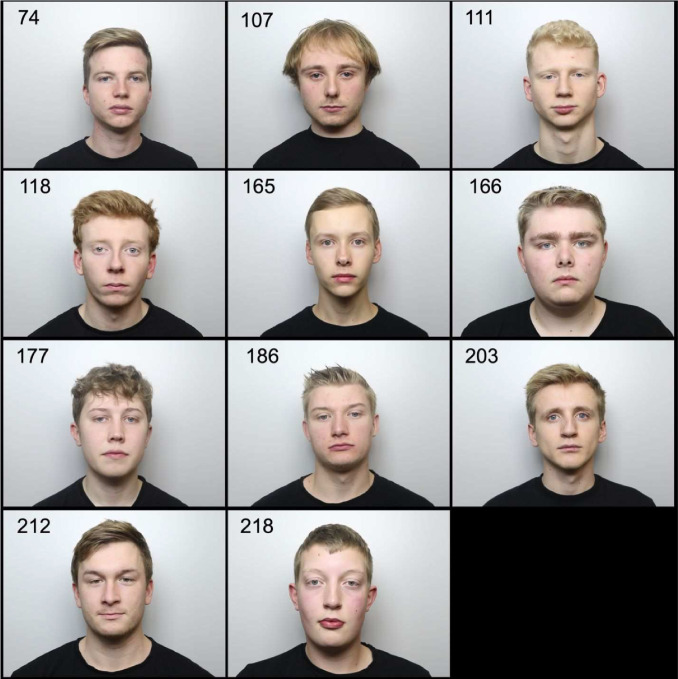
White male blonde

**Fig. 13:**
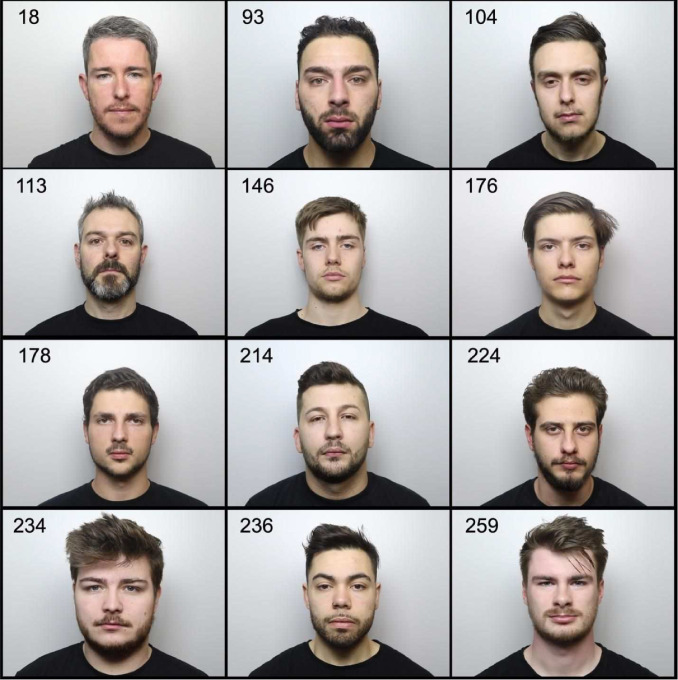
White male facial hair

**Fig. 14:**
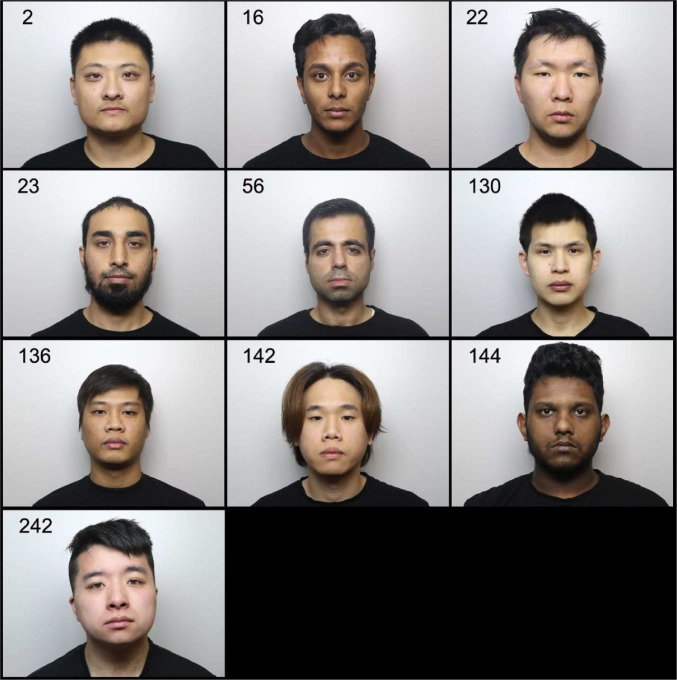
Asian male

**Fig. 15:**
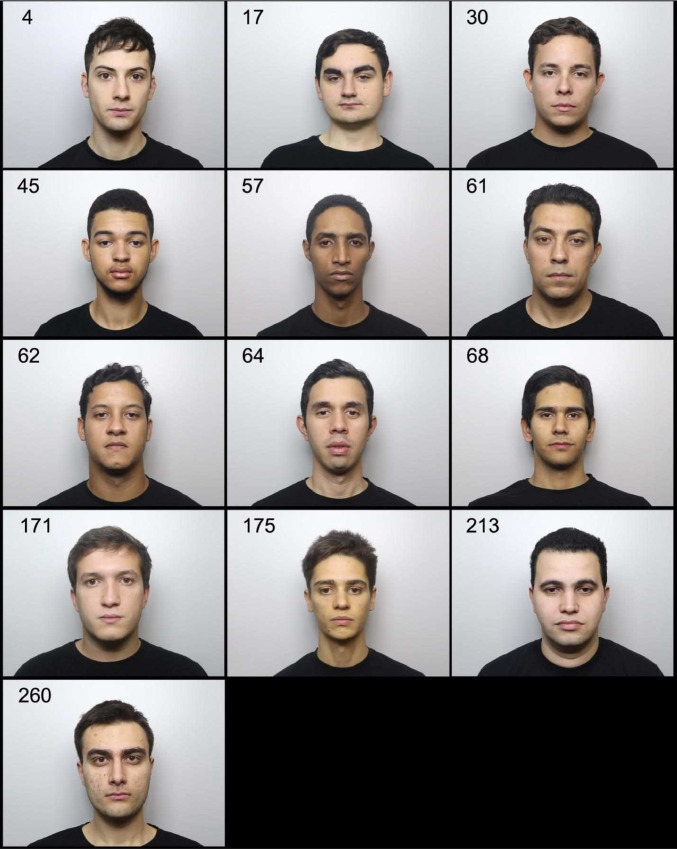
Multiple races male

**Fig. 16:**
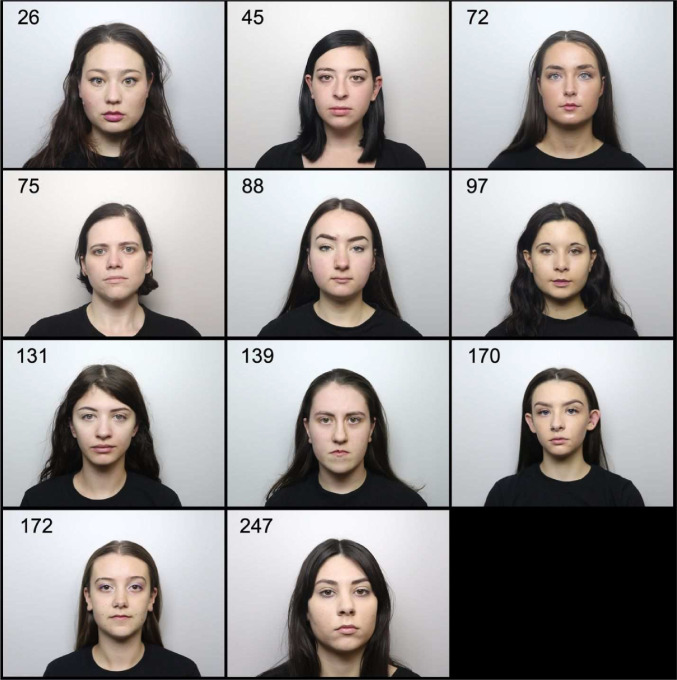
White female black hair

**Fig. 17:**
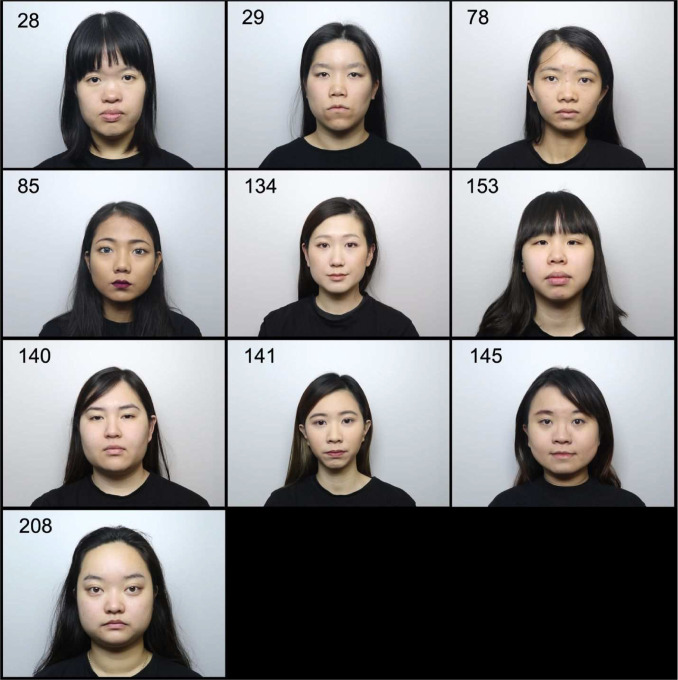
Asian female

**Fig. 18:**
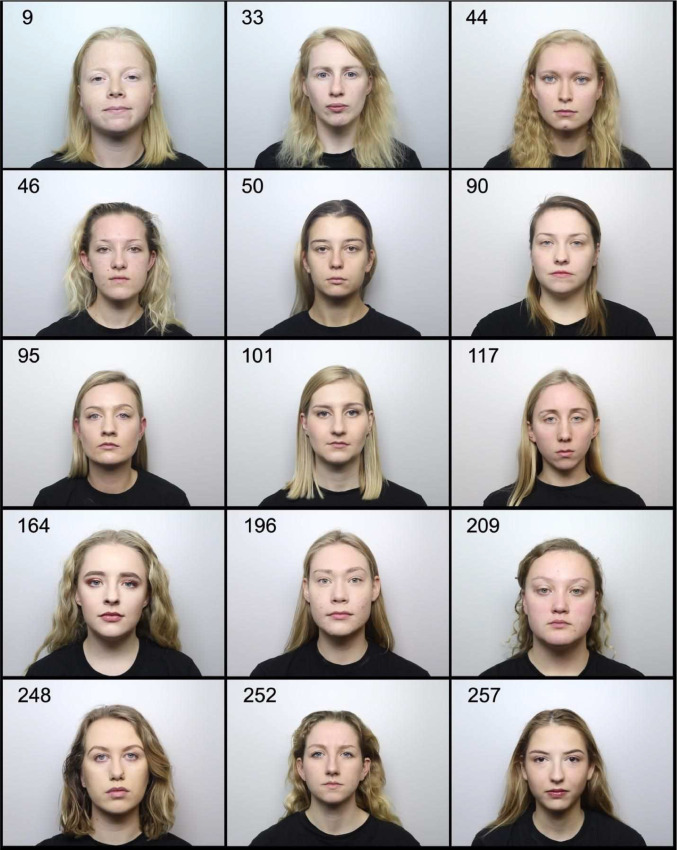
White female blonde 1

**Fig. 19:**
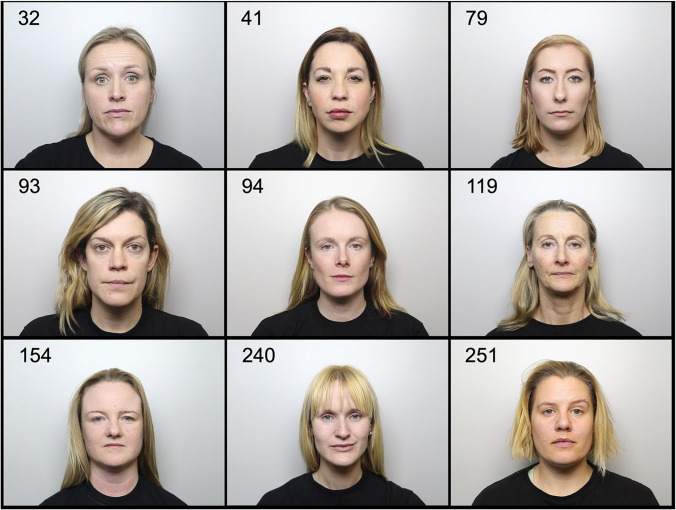
White female blonde 2

Figure 3 depicts the association between eyewitness confidence and PPV across the three lineup sizes. Panel A shows PPV with no correction to the false-alarm rate, which assumes any false alarm would implicate an innocent suspect and is known in the literature as a calibration curve (Brewer & Wells, 2006). Panel B shows PPV after correcting the false-alarm rate for the nominal size of the lineup (e.g., divide by six for six-member lineups). The nominal size correction assumes the risk to an innocent suspect is no greater than to the average filler. Plotting the nominal-size-corrected estimate of PPV by confidence is known as a confidence accuracy characteristic curve (Mickes, 2015).

#### Trial 5 and 6

Table 2 in the Appendix reports disaggregated data for the 24 lineups used on Trials 5–6, which always included six members in the lineup. In these trials, the average hit rate in culprit-present lineups was 61.7% [59.1, 64.2] and the average false-alarm rate in culprit-absent lineups was 23.9% [21.7, 26.2].

## Discussion

In our first comprehensive test of the database, we found that lineup size affected correct identifications in culprit-present lineups and mistaken identifications in culprit-absent lineups. In terms of the accuracy of eyewitness decisions, six-member lineups outperformed 9- and 12-member lineups. This finding is consistent with previous research (Juncu & Fitzgerald, 2021). However, eyewitness accuracy is not the most relevant measure for evaluating the utility of a procedure (Smith et al., 2020; Yang & Smith 2023). Lineups typically only include one suspect who faces the risk of conviction. Assuming that the lineup is unbiased and has only one suspect, the risk to the innocent suspect was lowest in 12-member lineups, albeit not significantly lower than in the 6- and 9-member lineups. Similarly, when we estimated the accuracy of suspect identifications (i.e., PPV), we found that lineup size had no effect.

Although larger lineups were associated with greater lineup fairness, we used an unconventional method of selecting culprits that have implications for the generalizability of this finding. To increase the number of stimuli tested, we randomly selected culprits who broadly matched a description of the lineup members. This means that contrary to a typical eyewitness experiment, where participants all witnessed the same culprit committing the crime and would have either the same or at least vaguely similar memories of the culprit, participants in our eyewitness experiment saw different culprits and were looking for different people in the lineup. Thus, although we found that the lineups had at least four plausible lineup members, the fairness of the same lineups we tested could be substantially different if only one culprit is used and that culprit happens to bear a strong resemblance to certain lineup members. This can be mitigated by using the similarity index that we produced to select culprits for future eyewitness experiments.

## General discussion

Using the eyewitness lineup paradigm, researchers have discovered social and cognitive factors associated with lineup identification accuracy (e.g., Brewer & Wells, 2011; Kovera & Evelo, 2017; Wixted et al., 2015). These research findings have led to important legal reforms for protecting innocent suspects from wrongful convictions (e.g., Wells et al., 1998, 2020). Some have even referred to the study of eyewitness identification as the “gold standard” of applied legal research (e.g., Albright & Garrett, 2022; Newirth & Scheck, 2014). We created the Eyewitness Lineup Identity (ELI) database to further support the science of eyewitness identification.

The ELI database can be used to conduct controlled eyewitness identification experiments. In our experiment, the lineups produced average true-positive identification rates of 50–60% and average false-positive identification rates of 30–40% (Fig. 1). These averages are consistent with those reported in previous eyewitness ID experiments. A critical advantage of the ELI database over existing face databases is that we recorded crime videos for each identity. This allows researchers to test performance using a variety of culprits and lineups, which reduces the risk of stimulus-specific effects and is beneficial for both the internal and external validity of the experiment (Wells & Windschitl, 1999).

The database is suitable for studying many but not all variables that are relevant to eyewitness identification. Although the database is relatively large compared to the stimulus sets used in previous eyewitness identification experiments, a set of 231 identities is not sufficient for creating lineups for any and all distinguishable groups of people. We were able to produce lineups with a reasonable amount of difficulty for some groups, but not all age groups are represented in the database, and many racial groups are also underrepresented.

The stimulus set is well suited for studying manipulations of the lineups as we did with lineup size, but the lack of variability in the content of the crime videos limits the types of eyewitness experiences that can be simulated. The crime videos in the ELI database are simple and depict a culprit always performing the same action for the same amount of time. Thus, the ELI crime videos are incapable of creating memories for more complicated witnessing events. For example, because the crime was always a theft and did not involve a weapon, the database cannot be used for studying the weapon-focus effect.

The ELI crime videos also have minimal distractions and provide relatively ideal viewing conditions. This may not be representative of most real-world crimes, and performance in research with ELI stimuli may not generalize to applied settings with less optimal conditions. Therefore, we warn against overreliance on the ELI database and encourage the production of additional eyewitness lineup identity databases.

Future databases should aim to expand the types of stimuli that are available for eyewitness research. One recommendation is to increase the realism of the stimuli, such as the recent development of 360° videos and 3D lineup images (Kruse & Schweinberger, 2023). It may also be fruitful to increase the diversity of crime events in future databases, as thefts are used in the majority of eyewitness studies. Finally, creating stimuli for a wider range of demographic groups is critical.

A further consideration is that after the database becomes available to the research community, the stimuli will become known to many of the professional research participants on crowdsourcing platforms, such as MTurk. Remote participation in eyewitness studies on these platforms has been on the rise (Kovera & Evelo, 2021). We have made the ELI database available to the research community, and it is currently the only available database with large numbers of crime videos and mugshots, so we do not currently recommend using it on widely used platforms like Mturk. Doing so would pose a nontrivial risk of unpredictable results due to the possibility of some participants having prior exposure to the stimuli from other studies. We hope our work will encourage more researchers to create databases for research on eyewitness identification, so that alternatives are available and the risk of prior familiarity with the ELI database becomes less of a concern.

## Conclusion

We created the ELI database to accelerate the science of eyewitness identification. The database contains crime videos for over 200 identities, which will facilitate stimulus sampling and reduce the risk of stimulus-specific effects. To increase the transparency of methods in eyewitness identification research, we obtained consent to include the stimuli in academic publications. We also tested 72 lineups constructed from the database in an eyewitness experiment. This experiment produced outcomes similar to previous research and can guide stimulus selection in future experiments. We conclude that the ELI database would be suitable for studying many research questions in the field of eyewitness identification.

## Data Availability

The Eyewitness Lineup Identity (ELI) database materials are accessible by request at https://osf.io/vrj3u for academic research that has been approved by a research ethics committee. Data for the eyewitness experiment are publicly accessible at https://osf.io/vrj3u.

## Appendix

Tables 1 and 2 report response rates for the 72 lineups tested in the eyewitness experiment. Lineups are labeled as [Race]_[Gender]_[LineupSize][LineupVersion]. For example, W_M_12L2 is White, male, 12-member lineup, Version 2. Identity numbers in Table 1 correspond with the numbers in Figs. 4, 5, 6, 7, 8, 9, 10, 11. Identity numbers in Table 2 correspond with the numbers in Figs. 4, 5, 6, 7, 8, 9, 10, 11, 12, 13, 14, 15, 16, 17, 18 and 19.

**Table 1:** Composition and performance of the 48 lineups in Trials 1–4

Lineup	Identities in the Lineup	Culprit present				Culprit absent		
		N	Hit	FA	Miss	N	FA	E
B_F_6L1	15, 40, 63, 76, 82, 103	58	.53	.29	.17	67	.36	3.27
B_F_6L2	76, 82, 103, 114, 138, 150	58	.67	.10	.22	74	.35	3.89
B_F_6L3	114, 138, 150, 192, 201, 210	59	.56	.15	.29	86	.29	3.65
B_F_6L4	14, 27, 35, 36, 58, 66	57	.53	.16	.32	77	.47	4.56
B_F_6L5	36, 58, 66, 69, 115, 147,	54	.44	.15	.41	66	.24	5.12
B_F_6L6	69, 115, 147, 149, 199, 226	64	.47	.14	.39	71	.44	2.71
MR_F_6L1	19, 25, 53, 60, 77, 80	58	.67	.02	.31	75	.16	3.60
MR_F_6L2	60, 77, 80, 87, 158, 191	57	.56	.09	.35	72	.24	5.07
MR_F_6L3	87, 158, 191, 204, 207, 231	58	.43	.09	.48	74	.20	4.09
MR_F_6L4	60, 77, 80, 81, 83, 137	58	.59	.09	.33	75	.19	3.92
MR_F_6L5	81, 83, 137, 155, 158, 207	56	.61	.04	.36	78	.26	3.70
MR_F_6L6	155, 158, 207, 231, 235, 237	60	.62	.07	.32	72	.17	4.80
W_F_Brunette_6L1	38, 89, 91, 105, 156, 161	56	.63	.14	.23	64	.28	3.95
W_F_ Brunette_6L2	105, 156, 161, 162, 182, 198	58	.72	.07	.21	79	.30	3.27
W_F_ Brunette_6L3	39, 162, 182, 198, 232, 239	58	.64	.16	.21	79	.30	4.36
W_F_ Brunette_6L4	37, 48, 92, 99, 160, 168	51	.49	.07	.19	76	.25	4.63
W_F_ Brunette_6L5	99, 160, 168, 184, 187, 193	55	.62	.11	.27	80	.31	4.08
W_F_ Brunette_6L6	184, 187, 193, 220, 223, 253	59	.64	.05	.31	79	.39	4.23
W_M_ Brunette_6L1	11, 34, 102, 124, 125, 174	61	.74	.05	.21	66	.29	4.06
W_M_ Brunette_6L2	124, 125, 174, 176, 205, 215	64	.63	.08	.30	63	.22	5.16
W_M_ Brunette_6L3	176, 205, 215, 221, 230, 233	66	.53	.12	.35	68	.19	4.33
W_M_ Brunette_6L4	13, 84, 112, 122, 127, 135	59	.71	.05	.24	74	.38	4.90
W_M_ Brunette_6L5	112, 127, 135, 167, 181, 197	60	.85	.05	.10	81	.32	2.96
W_M_ Brunette_6L6	167, 181, 197, 202, 205, 206	59	.53	.24	.24	73	.26	4.46
B_F_9L1	15, 40, 63, 76, 82, 103, 114, 138, 150	45	.47	.27	.27	52	.44	4.52
B_F_9L2	76, 82, 103, 114, 138, 150, 192, 201, 210	44	.59	.25	.16	56	.45	4.14
B_F_9L3	14, 27, 35, 36, 58, 66, 69, 115, 147	42	.45	.26	.29	52	.62	3.68
B_F_9L4	36, 58, 66, 69, 115, 147, 149, 199, 226	46	.41	.33	.26	53	.42	4.75
MR_F_9L1	19, 25, 53, 60, 77, 80, 87, 158, 191	44	.66	.11	.23	57	.28	6.10
MR_F_9L2	60, 77, 80, 87, 158, 191, 204, 207, 231	46	.67	.15	.17	52	.23	6.55
MR_F_9L3	60, 77, 80, 81, 83, 137, 155, 158, 207	44	.55	.07	.39	50	.30	4.09
MR_F_9L4	81, 83, 137, 155, 158, 207, 231, 235, 237	44	.68	.05	.27	54	.24	4.33
W_F_ Brunette_9L1	38, 39, 89, 91, 105, 156, 161, 162, 182	39	.67	.08	.26	56	.38	4.55
W_F_ Brunette_9L2	39, 105, 156, 161, 162, 182, 198, 232, 239	42	.48	.24	.29	56	.34	4.81
W_F_ Brunette_9L3	37, 48, 92, 99, 160, 168, 184, 187, 193,	44	.73	.09	.18	50	.54	7.22
W_F_ Brunette_9L4	99, 160, 168, 184, 187, 193, 220, 223, 253	42	.60	.21	.19	60	.53	6.24
W_M_ Brunette_9L1	11, 34, 102, 124, 125, 174, 176, 205, 215	50	.66	.14	.20	52	.38	8.00
W_M_ Brunette_9L2	124, 125, 174, 176, 205, 215, 221, 230, 233	46	.57	.13	.30	50	.28	6.53
W_M_ Brunette_9L3	13, 84, 112, 122, 127, 135, 167, 181, 197	48	.75	.06	.19	54	.37	3.77
W_M_ Brunette_9L4	112, 127, 135, 167, 181, 197, 202, 205, 206	44	.64	.09	.27	47	.40	5.39
B_F_12L1	15, 40, 63, 76, 82, 103, 114, 138, 150, 192, 201, 210	97	.47	.27	.26	98	.49	6.19
B_F_12L2	14, 27, 35, 36, 58, 66, 69, 115, 147, 149, 199, 226	110	.45	.29	.25	86	.57	4.41
MR_F_12L1	19, 25, 53, 60, 77, 80, 87, 158, 191, 204, 207, 231	109	.54	.16	.30	97	.27	8.45
MR_F_12L2	60, 77, 80, 81, 83, 137, 155, 158, 207, 231, 235, 237	95	.53	.11	.37	94	.26	4.65
W_F_ Brunette_12L1	38, 39, 89, 91, 105, 156, 161, 162, 182, 198, 232, 239	92	.53	.17	.29	104	.45	8.21
W_F_ Brunette_12L2	37, 48, 92, 99, 160, 168, 184, 187, 193, 220, 223, 253	91	.57	.20	.23	105	.46	7.07
W_M_ Brunette_12L1	11, 34, 102, 124, 125, 174, 176, 205, 215, 221, 230, 233	104	.58	.15	.27	96	.34	9.00
W_M_ Brunette_12L2	13, 84, 112, 122, 127, 135, 167, 181, 197, 202, 205, 206	106	.58	.24	.19	89	.42	6.37

Lineups are labeled as [Race]_[Gender]_[LineupSize][LineupVersion]. W = White. B = Black. MR = Multiple Races. M = Male. F = Female. Hits = correct identification rate. FA = false-alarm/mistaken identification rate. Miss = false rejection of lineup rate. E = Effective size of lineup.

**Table 2:** Composition and performance of the 24 lineups in Trials 5–6

Lineup	Identities in the lineup	Culprit present				Culprit absent		
		N	Hit	FA	Miss	N	FA	E
A_M_6L1	2, 16, 56, 136, 142, 228	68	.78	.00	.22	63	.14	3.52
A_M_6L2	2, 22, 130, 136, 142, 242	66	.67	.05	.29	66	.11	2.88
A_M_6L3	16, 23, 56, 136, 144, 228,	63	.70	.02	.29	73	.05	1.60
A_F_6L1	28, 29, 78, 140, 141, 145	66	.58	.08	.35	67	.28	3.50
A_F_6L2	78, 85, 134, 145, 153, 208	72	.68	.06	.26	64	.28	5.59
A_F_6L3	29, 78, 134, 140, 141, 145	68	.54	.13	.32	56	.32	3.86
MR_M_6L1	30, 61, 62, 64, 68, 70	63	.54	.10	.37	65	.26	1.94
MR_M_6L2	4, 17, 30, 171, 213, 260	61	.39	.15	.46	63	.22	3.77
MR_M_6L3	43, 57, 62, 64, 68, 175	64	.69	.06	.25	74	.14	4.17
W_F_BlackHair_6L1	45, 97, 131, 170, 172, 247	67	.57	.12	.31	63	.30	4.46
W_F_BlackHair_6L2	75, 88, 131, 139, 170, 172	69	.62	.03	.35	63	.21	4.33
W_F_BlackHair_6L3	26, 45, 72, 97, 131, 247	63	.54	.10	.37	63	.17	4.17
W_F_Blonde_6L1	9, 50, 95, 101, 117, 196	60	.57	.23	.20	73	.38	4.17
W_F_Blonde_6L2	33, 44, 46, 90, 209, 252	65	.48	.14	.38	64	.33	4.05
W_F_Blonde_6L3	95, 101, 117, 164, 248, 257	62	.63	.15	.23	69	.30	4.96
W_F_Blonde_6L4	32, 41, 79, 93, 94, 154	73	.59	.10	.32	63	.16	1.47
W_F_Blonde_6L5	32, 94, 119, 154, 240, 251	69	.57	.01	.42	65	.12	2.91
W_F_Blonde_6L6	32, 79, 93, 94, 154, 240	68	.71	.03	.26	61	.41	3.21
W_M_Blonde_6L1	111, 118, 166, 186, 203, 212	66	.67	.05	.29	69	.22	3.26
W_M_Blonde_6L2	107, 166, 177, 203, 212, 218	41	.27	.17	.56	89	.24	2.38
W_M_Blonde_6L3	74, 111, 118, 165, 186, 218	66	.79	.05	.17	61	.41	3.14
W_M_FacialHair_6L1	18, 104, 146, 178, 234, 259	65	.55	.08	.37	62	.23	3.63
W_M_FacialHair_6L2	96, 113, 214, 224, 234, 236,	68	.75	.06	.19	64	.20	2.86
W_M_FacialHair_6L3	104, 146, 178, 214, 234, 259	64	.56	.03	.41	70	.19	4.12

Lineups are labeled as [Race]_[Gender]_[LineupSize][LineupVersion]. W = White. A = Asian. MR = Multiple Races. M = Male. F = Female. Hits = correct identification rate. FA = false alarm/mistaken identification rate. Miss = false rejection of lineup rate. E = Effective size of lineup

The mugshots that were tested in the eyewitness experiment are presented in Figs. 4, 5, 6, 7, 8, 9, 10, 11, 12, 13, 14, 15, 16, 17, 18 and 19. The people depicted in the database are actors, not actual culprits or lineup members in real criminal cases. Mugshots in the database were created with a recording booth on loan from the Video Identification Parade Electronic Recording (VIPER) Bureau, West Yorkshire Police, England. Images in the database were not quality assured by the VIPER Bureau, and the authors accept full responsibility for their quality.

## Footnotes

1 For Identity 149, the crime video and mugshot were not recorded on the same day. The mugshot for Identity 149 depicts her with straight hair. In the experiment, we used a crime video depicting Identity 149 with braided hair, which was recorded approximately 2 weeks after the mugshot was recorded. The ELI database also contains another crime video that depicts Identity 149 with straight hair, which was recorded 2 days after the mugshot was taken. This additional crime video has a different plot in which Identity 149 uses a computer and abruptly leaves, and the words “cyber attack” are displayed on the computer screen.

2 Identity #70 (*n* = 35) was tested in the experiment but has since been removed from the database after we increased the level of consent required to be included in the database. This identity is excluded from the Identities data file. Identity #232 appeared in two pools. To avoid confusion, the Identities file only includes data for one of the pools (White women with dark hair) and excludes trials for lineups from the other pool (*n* = 54). The excluded data for #70 and #232 are included in the “Eyewitness” data file.

## Code availability

No code is provided.
